# Choline in Adolescent Pregnancy: The Impact on Fetal Brain Development and Long-Term Cognitive Outcomes of Offspring

**DOI:** 10.3390/medicina61112057

**Published:** 2025-11-18

**Authors:** Abdul Jabar Khudor, Marius Alexandru Moga, Oana Gabriela Dimienescu, Andrada Camelia Nicolau, Cristian Andrei Arvătescu, Mircea Daniel Hogea, Natalia Ciobanu

**Affiliations:** 1 Medicine PhD School, Transilvania University of Brasov, 500036 Brașov, Romania; 2 Faculty of Medicine, Transilvania University of Brasov, 500036 Brașov, Romania

**Keywords:** choline, adolescent pregnancy, brain development, placental transport, neurological development, prenatal nutrition

## Abstract

Pregnancy in adolescence represents a major nutritional challenge, with competing demands between maternal development and fetal growth. Choline is the essential nutrient with a critical role for fetal brain development and exhibits distinct metabolic patterns in pregnant adolescents aged 15–19 years compared to adult pregnant women. This narrative review examines the specific impact of choline status on fetal neurodevelopment in adolescent pregnancies. A comprehensive literature review was conducted using PubMed and Web of Science databases from 2000 to 2025, focusing on choline metabolism, placental transport mechanisms, and neurodevelopmental outcomes in adolescent pregnancy. Adolescent pregnant women demonstrate reduced choline clearance (0.8 ± 0.2 vs. 1.2 ± 0.3 mL/min/kg), decreased choline kinase activity (25–30% reduction), and reduced placental transporter expression (CTL1 reduced by 15–20%) compared to adults. These metabolic differences result in maternal–fetal competition for limited choline resources, potentially compromising fetal brain development during critical neurodevelopmental windows. The consequences include increased risk of neural tube defects, altered hippocampal development, and long-term cognitive impairments in offspring. Adolescent pregnancy creates a unique biochemical environment that may predispose to choline deficiency with lasting neurodevelopmental consequences, and current supplementation guidelines do not address adolescent-specific needs, pointing out the urgent requirement for appropriate age recommendations and targeted interventions to optimize maternal and fetal outcomes in this vulnerable population.

## 1. Introduction

Adolescent pregnancy remains a significant global health challenge, affecting approximately 21 million girls aged 15–19 years annually [1]. These young mothers cope with unique physiological demands as their own growth and development continue while simultaneously supporting fetal development. Among the critical nutritional factors during pregnancy, choline has emerged as particularly important for optimal fetal brain development and long-term cognitive outcomes [2,3,4].

Choline was recognized as an essential nutrient by the Institute of Medicine in 1998 and plays multiple roles in human physiology [5]. During pregnancy, choline serves as a precursor for phosphatidylcholine synthesis, acetylcholine production and methylation reactions that influence gene expression and fetal programming [6,7]. The developing fetal brain exhibits particularly high choline requirements, with concentrations in fetal plasma exceeding maternal levels during the third trimester [8,9].

Adolescent pregnancy is different from adult pregnancy because of the ongoing maternal brain development that continues until approximately 25 years of age [10], therefore two developing nervous systems compete for the same nutritional resources. Furthermore, adolescents typically have lower dietary choline intake, reduced hepatic choline reserves, and different metabolic patterns compared to adult women [11,12].

Recent studies have shown that pregnant adolescents exhibit distinct choline transport mechanisms, altered enzyme activities, and modified placental function that may compromise fetal choline availability [13,14,15]. These findings have profound implications for fetal neurodevelopment, as choline deficiency during critical periods can result in permanent alterations to brain structure and function [16,17,18].

The objectives of this narrative review are to examine the aspects of choline metabolism in adolescent pregnancy, analyze placental choline transport mechanisms and their limitations in young mothers, evaluate the pathophysiological consequences of choline deficiency on fetal brain development, assess current evidence regarding cognitive outcomes in offspring of adolescent mothers, and provide evidence-based recommendations for clinical practice and future research directions. This narrative review was conducted through systematic searches of PubMed and Web of Science databases covering the period from 2000 to 2025. Search terms included combinations of “choline,” “adolescent pregnancy,” “teenage pregnancy,” “fetal brain development,” “cognitive outcomes,” “placental transport,” and “neurological development.” We prioritized human studies but also included relevant animal studies that provided mechanistic insights. Articles were selected based on relevance to choline metabolism, placental function, and neurodevelopmental outcomes in adolescent pregnancies. Both peer-reviewed original research articles and authoritative review articles were included.

### Evolutionary and Developmental Context of Adolescent Pregnancy

From an evolutionary developmental biology perspective, adolescent pregnancy represents a complex paradox in modern medicine. Historically, human females evolved to achieve reproductive maturity and conceive during ages 15–20 years, coinciding with what evolution optimized as the period of peak biological capacity for pregnancy and childbirth [19,20]. The evolutionary rationale for early reproduction in human history was clear: in environments characterized by high mortality risk, early fecundity maximized the likelihood of reproduction before death. Studies of contemporary hunter-gatherer populations demonstrate this adaptive strategy, with first reproduction occurring at young ages and short life expectancies representing an evolutionary adaptation to high-risk environments. However, current epidemiological data reveal a secular trend toward even earlier pubertal onset than historical patterns from recent centuries. The age of menarche has declined by approximately 4 years over the past 150 years in industrialized societies. This acceleration has been attributed to multiple factors including improved nutrition, reduced infectious disease burden, decreased physical labor demands, and potentially environmental endocrine-disrupting chemicals. Critically, contemporary research demonstrates that not only nutritional abundance, but also psychosocial environmental stressors can advance pubertal timing through epigenetic mechanisms. Belsky et al.’s evolutionary theory of socialization proposes that familial psychosocial stress (e.g., harsh parenting, marital conflict, father absence) fosters accelerated pubertal development as an adaptive reproductive strategy. In uncertain or stressful environments, earlier maturation may enhance reproductive fitness by reducing the risk of death before reproduction and enabling more reproductive cycles over a lifetime. This framework challenges the conventional view of early puberty as exclusively pathological, instead recognizing it as a developmentally plastic response to environmental cues. The unique nutritional challenge of modern adolescent pregnancy emerges from a critical disconnect between biological reproductive capacity and ongoing neurodevelopmental needs. While evolution optimized female physiology for reproduction during mid-to-late adolescence, several key developmental processes remain incomplete in contemporary adolescent pregnancies:

- maternal brain development continues until approximately 25 years of age, with the prefrontal cortex among the last regions to mature [20]. This ongoing neural maturation creates direct competition between maternal and fetal brains for essential nutrients, particularly those required for membrane synthesis, neurotransmitter production, and myelination.
- modern adolescents in developed countries typically experience menarche at ages when peak linear growth has not yet been achieved, creating simultaneous demands for nutrients to support both continued maternal skeletal growth and fetal development. This differs from the evolutionary pattern, where menarche typically occurred near the completion of growth.
- contemporary adolescents often have lower pre-pregnancy nutritional reserves compared to adult women, particularly for nutrients like choline that require hepatic storage. Studies demonstrate that adolescents have approximately 40–50% lower liver choline concentrations compared to adults, limiting the ability to mobilize reserves during pregnancy.

This evolutionary-developmental mismatch particularly affects nutrient partitioning for choline, as both maternal and fetal nervous systems simultaneously require substantial choline resources during overlapping critical developmental windows. Understanding this evolutionary context is essential for developing appropriate nutritional interventions that address the unique metabolic demands of adolescent pregnancy, recognizing that modern adolescent mothers face unprecedented physiological challenges that evolution did not anticipate or optimize.

## 2. Choline Metabolism and Biochemical Pathways

### 2.1. The Kennedy Pathway: Phospholipid Synthesis

The Kennedy pathway represents the primary route for phosphatidylcholine (PC) biosynthesis, accounting for approximately 70–80% of total PC production in cells [21]. This pathway begins with choline uptake via specific transporters, followed by phosphorylation to phosphocholine by choline kinase (CK), conversion to CDP-choline by CTP: phosphocholine cytidylyltransferase (CCT), and final incorporation into phosphatidylcholine by CDP-choline:1,2-diacylglycerol cholinephosphotransferase [22,23].

In adolescent pregnancy, several key differences emerge in Kennedy pathway regulation. Choline kinase α (CHKA) activity is reduced by 25–30% compared to adult pregnant women, potentially limiting the initial step of PC synthesis [24]. This reduction is particularly significant given the increased PC demands during fetal brain development, where PC serves as the predominant membrane phospholipid [25,26]. The rate-limiting enzyme CCT also exhibits altered regulation in adolescents. While CCT activity typically increases during normal pregnancy, this upregulation is blocked in adolescents, resulting in only a 20–30% increase [27,28,29]. This enzymatic response may contribute to the reductions in maternal plasma PC concentrations in pregnant adolescents compared to adults [29]. The rate-limiting enzyme CCT also exhibits altered regulation in adolescents. While CCT activity typically increases during normal pregnancy, this upregulation is blunted in adolescents, resulting in only a 20–30% increase compared to 40–60% in adults [30,31]. The reduced CCT activity in adolescents appears to result from several factors: (1) hormonal immaturity, particularly lower estrogen levels which normally upregulate CCT expression through estrogen response elements in gene promoter regions [6,30,31]; (2) ongoing competition for lipid substrates between maternal growth and pregnancy demands, limiting the availability of diacylglycerol required for phosphatidylcholine synthesis; (3) reduced expression of fatty acid binding proteins that facilitate CCT membrane translocation and activation; and (4) altered lipid membrane composition in adolescent hepatocytes, affecting CCT activation which requires interaction with anionic phospholipids. This enzymatic limitation may contribute to the reduced maternal plasma phosphatidylcholine concentrations observed in pregnant adolescents compared to adults [6,26,31].

### 2.2. Acetylcholine Synthesis Pathway

The synthesis of acetylcholine (ACh) from choline represents a critical pathway for neurotransmitter production, particularly important during fetal brain development [32,33,34]. Choline acetyltransferase (ChAT) catalyzes the condensation of choline with acetyl-CoA to form ACh, which is essential for cholinergic neurotransmission and proper neural development [33,34]. During pregnancy, fetal brain ChAT activity increases dramatically, particularly during the second and third trimesters when cholinergic neurons undergo rapid development and synaptogenesis [35]. In adolescent pregnancies, maternal ChAT activity shows only modest increases (20–25%) compared to the 40–50% increase observed in adult pregnancies [36]. This differential response may limit acetylcholine availability for both maternal and fetal neural functions.

The competition for choline between PC synthesis and ACh production becomes particularly relevant in adolescent pregnancy. Unlike adults, who can mobilize hepatic choline reserves, adolescents have limited stored choline (approximately 40–50% less than adults), making them more susceptible to functional choline deficiency when demands increase [37,38].

### 2.3. Betaine Pathway: Methylation Reactions

The betaine pathway represents an alternative route for choline utilization, involving oxidation to betaine and subsequent donation of methyl groups for homocysteine remethylating to methionine [39]. This pathway is particularly important for maintaining adequate methylation capacity during pregnancy, as methionine serves as the precursor for S-adenosylmethionine (SAM), the universal methyl donor [40].

In adolescent pregnancy, betaine pathway activity shows distinct patterns compared to adults. Choline dehydrogenase activity, the first enzyme in betaine synthesis, is paradoxically increased in adolescents, potentially representing a compensatory mechanism for reduced Kennedy pathway flux [41]. However, this increase may divert choline away from essential PC and ACh synthesis, creating an internal competition for limited choline resources [42].

The methylation demands of pregnancy are particularly high during the first trimester when DNA methylation patterns are established in the developing embryo [43]. Adolescent mothers show reduced methylation capacity, as evidenced by elevated plasma homocysteine concentrations and decreased SAM ratios compared to adult pregnant women [44,45].

### 2.4. Adolescent-Specific Metabolic Differences

**Figure 1:**
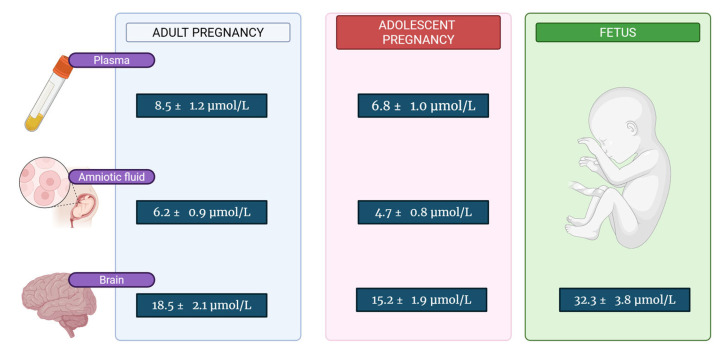
Summary of the Choline Concentrations in Maternal and Fetal Tissues.

Several key metabolic differences distinguish choline handling in adolescent versus adult pregnancy. First, hepatic choline storage is significantly reduced in adolescents, with liver choline concentrations approximately 40–50% lower than adults [46,47]. This reduced storage capacity limits the ability to mobilize choline during periods of increased demand. Choline clearance rates are substantially lower in adolescents (0.8 ± 0.2 mL/min/kg) compared to adults (1.2 ± 0.3 mL/min/kg), suggesting altered renal handling or tissue uptake mechanisms [47]. This reduced clearance may represent an adaptive mechanism to conserve choline but could also indicate impaired cellular uptake. Choline concentrations in maternal and fetal tissues are exemplified in Table 1 and Figure 1.

The expression of key enzymes in choline metabolism shows age-dependent patterns. PEMT (phosphatidylethanolamine N-methyltransferase) activity, which provides an alternative pathway for PC synthesis, is reduced by approximately 30% in adolescents, further limiting PC production capacity [48,49,50,51].

**Table 1:** Choline Concentrations in Maternal and Fetal Tissues (μmol/L).

Tissue/Fluid	Adult Pregnancy	Adolescent Pregnancy	Fetus (Third Trimester)	Ref.
Maternal Plasma	8.5 ± 1.2	6.8 ± 1.0	-	[44,45]
Fetal Plasma	22.1 ± 3.5	18.3 ± 2.8	20.2 ± 3.1	[52,53]
Amniotic Fluid	6.2 ± 0.9	4.7 ± 0.8	-	[54]
Maternal Brain	18.5 ± 2.1	15.2 ± 1.9	-	[55]
Fetal Brain	35.8 ± 4.2	28.9 ± 3.6	32.3 ± 3.8	[56,57]

## 3. Placental Choline Transport Mechanisms

### 3.1. Choline Transporter-like Protein 1 (CTL1/SLC44A1)

CTL1 represents the primary choline transporter in human placenta, exhibiting intermediate affinity (Km ~5–20 μM) and high capacity for choline transport [58]. Located predominantly in the syncytiotrophoblast microvillous membrane, CTL1 facilitates the initial uptake of choline from maternal circulation [26]. This transporter shows Na^+^-independent, bidirectional transport characteristics, making it particularly suitable for maintaining maternal–fetal choline gradients [59].

In adolescent pregnancies, CTL1 expression is significantly reduced (15–20% decrease) compared to adult pregnancies [60]. This reduction appears to be related to the overall immaturity of placental development in young mothers, as CTL1 expression normally increases throughout gestation [61]. The functional consequence is a reduced capacity for maternal choline uptake, potentially limiting fetal choline availability during critical developmental periods.

**Figure 2:**
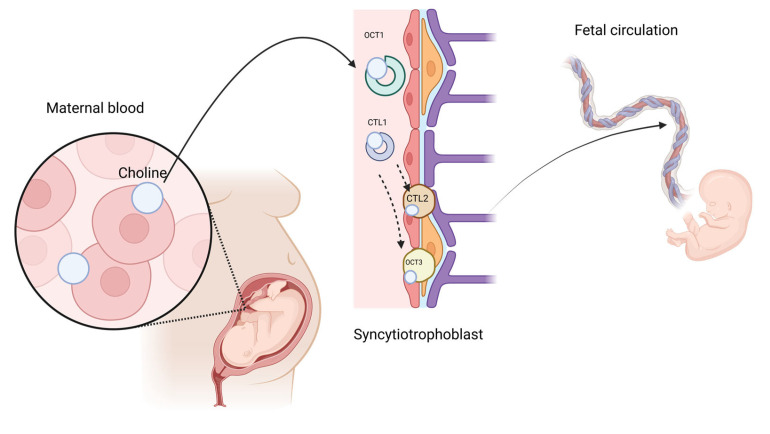
Choline transport mechanisms through placenta.

Regulation of CTL1 is complex and involves multiple factors including hormonal influences, nutritional status, and epigenetic modifications [62]. Estrogen and progesterone, which show different patterns in adolescent versus adult pregnancy, can influence CTL1 expression through estrogen response elements in the promoter region [62]. The altered hormonal milieu in adolescent pregnancy may contribute to reduced CTL1 expression and function. In Figure 1 is pointed out the localization and function of choline transporters CTL1, CTL2, and OCT1/OCT3 across the placental barrier, with emphasis on transport limitations in adolescent pregnancies. Figure 2 summarizes the placental choline transport mechanism. 

### 3.2. Choline Transporter-like Protein 2 (CTL2/SLC44A2)

CTL2 serves as a secondary choline transport system with lower affinity (Km ~50–100 μM) but broader substrate specificity [63]. Located primarily in fetal capillary endothelium, CTL2 may function as a regulatory mechanism to prevent excessive choline accumulation in fetal tissues [64]. The transport capacity of CTL2 is generally lower than CTL1, contributing approximately 20–30% of total placental choline transport [65].

Adolescent pregnancies show preserved CTL2 expression, suggesting that this transporter may serve as a compensatory mechanism when CTL1 function is impaired [66]. The lower affinity of CTL2 means that significant compensation requires elevated choline concentrations, which may not be achievable in adolescents with limited choline reserves [67].

### 3.3. Organic Cation Transporters (OCT1, OCT3)

The organic cation transporter family, particularly OCT1 (SLC22A1) and OCT3 (SLC22A3), contributes to placental choline transport, although with lower specificity and efficiency compared to CTL transporters [68]. These transporters exhibit polyspecific substrate recognition and may be more important for the transport of choline metabolites such as acetylcholine [69].

OCT3 expression is particularly relevant as it shows significant developmental regulation and can transport both choline and acetylcholine bidirectionally [70]. In adolescent pregnancies, OCT3 expression appears to be maintained or even slightly increased, possibly representing an adaptive response to reduced CTL1 function [71].

### 3.4. Transport Limitations in Adolescent Pregnancy

Several factors contribute to impaired choline transport in adolescent pregnancies. The overall placental development may be delayed or suboptimal in very young mothers (15–16 years), resulting in reduced transporter expression and function [72], the competing nutritional demands of ongoing maternal growth may alter placental nutrient prioritization [73] and hormonal immaturity in adolescent pregnancies can affect transporter regulation. The hypothalamic–pituitary–adrenal axis is still developing in adolescents, and cortisol patterns differ significantly from adults, potentially influencing transporter gene expression [74]. Also, epigenetic factors, including DNA methylation patterns that regulate transporter genes, may be altered in adolescent pregnancies [75]. The key enzymes in choline metabolism during pregnancy are summarized in Table 2. 

Placental choline transport capacity demonstrates significant regulation, with transporter expression and activity increasing throughout gestation. However, adolescent pregnancy is characterized by several factors that may limit optimal choline transport efficiency.

Placental development in adolescent pregnancies often demonstrates delayed maturation, potentially affecting transporter expression patterns [2,85], while competing maternal developmental needs during adolescence may influence the allocation of resources for placental transporter synthesis. Studies have shown 15–20% reduced CTL1 expression in placentas from adolescent mothers compared to adult controls, potentially compromising maternal–fetal choline transfer. Table 3 summarizes the functional characteristics of placental choline transporters.

### 3.5. Mechanistic Basis of Transport Limitations in Adolescent Pregnancy

Several interconnected factors contribute to impaired choline transport capacity in adolescent pregnancies, creating a metabolically induced deficiency state that extends beyond simple dietary inadequacy. These limitations arise from fundamental differences in placental development, endocrine regulation, and nutrient prioritization that distinguish adolescents from adult pregnancy.

#### 3.5.1. Placental Developmental Immaturity

The placenta in adolescent pregnancy demonstrates fundamental structural and functional differences compared to adult pregnancy [90]. Morphological studies reveal that adolescent placentas exhibit delayed maturation characterized by reduced syncytiotrophoblast surface area and decreased villous branching complexity. This structural immaturity directly constrains the spatial distribution and functional density of nutrient transporters. The microvillous membrane surface area—where CTL1 is predominantly expressed—shows reduction in placentas from mothers aged 15–17 years compared to those aged 20–35 years [90]. This reduction in functional surface area compounds the observed decrease in CTL1 protein expression per unit membrane area, resulting in an estimated overall reduction in maternal-to-fetal choline transport capacity. The placental vasculature in adolescent pregnancies also demonstrates reduced angiogenic maturation, with decreased vessel branching density and smaller vessel caliber in tertiary villi. This vascular limitation may impair the blood flow-dependent aspects of choline delivery, particularly under conditions of increased metabolic demand.

#### 3.5.2. Altered Endocrine Regulation

The hormonal milieu of adolescent pregnancy differs substantially from adult pregnancy in ways that directly affect transporter expression and function [91,92]. Three key endocrine differences are particularly relevant:

- placental growth hormone variant (GH-V) secretion follows a distinct pattern in adolescent versus adult pregnancy. GH-V normally increases exponentially from weeks 15–37 of gestation and plays a crucial role in upregulating placental nutrient transporter expression through STAT5 signaling pathways. Adolescent pregnancies may demonstrate altered GH-V patterns, potentially limiting transporter upregulation during critical developmental windows.
- the estrogen-to-progesterone ratio shows age-dependent variations that influence CTL1 expression. CTL1 gene promoter analysis reveals functional estrogen response elements (EREs) that mediate transcriptional activation. Adolescent pregnancies exhibit altered estrogen: progesterone ratios, particularly in early pregnancy, which may affect the hormonal drive for CTL1 upregulation.
- leptin and adiponectin—adipokines that regulate placental nutrient transport—show distinct patterns in adolescent pregnancies [5]. Leptin normally increases throughout pregnancy and enhances amino acid transporter expression through mTOR pathway activation. Adolescent pregnant women may demonstrate different leptin concentrations adjusted for body fat percentage, potentially reflecting ongoing competition between maternal growth needs and pregnancy adaptations.

#### 3.5.3. Nutrient Prioritization and Maternal-Placental Competition

Perhaps the most unique aspect of adolescent pregnancy is the triple competition for nutrients among ongoing maternal growth, placental development, and fetal growth [91]. This differs fundamentally from adult pregnancy, where maternal growth is complete and competition exists only between placental and fetal tissues. Amino acid transporter studies provide insight into this competitive dynamic. System A amino acid transporters (SNAT1, SNAT2, SNAT4) show reduced expression and activity in placentas from pregnant adolescents compared to adults [91]. Importantly, the degree of transporter downregulation correlates inversely with maternal height velocity, suggesting that placentas in actively growing adolescents prioritize maternal nutrient delivery over placental-to-fetal transfer. Choline kinase activity—the first enzyme in the Kennedy pathway for phosphatidylcholine synthesis—may be elevated in maternal liver and skeletal muscle of pregnant adolescents compared to pregnant adults, suggesting enhanced maternal tissue retention of choline, potentially at the expense of placental transfer. The placenta itself has substantial choline requirements for its own growth and membrane synthesis. Placental choline kinase activity may be higher in adolescents compared to adult pregnancies, suggesting that a greater proportion of maternal choline is consumed by the placenta rather than transferred to the fetus.

## 4. Pathophysiology of Choline Deficiency in Adolescent Pregnancy

### 4.1. Maternal–Fetal Competition for Choline Resources

The pathophysiology of choline deficiency in adolescent pregnancy is fundamentally different from adult pregnancy due to the unique biological background of two developing nervous systems competing for limited choline resources [93]. Unlike adult women, whose brain development is complete, adolescent mothers continue neural maturation, particularly in prefrontal cortex regions, until approximately 25 years of age [94,95].

**Figure 3:**
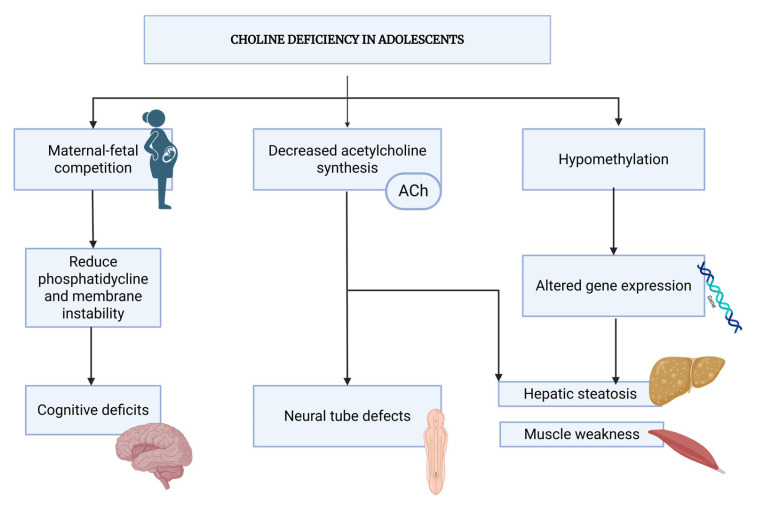
Pathophysiological Cascade of Choline Deficiency.

Figure 3 illustrates the progression from choline deficiency in adolescent pregnancy through various pathophysiological mechanisms to final outcomes affecting both maternal and fetal health.

This competition manifests at multiple levels: at the cellular level, both maternal and fetal tissues express high-affinity choline transporters, creating direct competition for circulating choline. The fetal brain typically maintains a concentration gradient over maternal plasma, but this gradient may be compromised in adolescent pregnancies with limited choline availability [96,97]. At the metabolic level, the ongoing myelination in adolescent maternal brains requires substantial phosphatidylcholine synthesis, directly competing with fetal demands [87]. Studies using choline tracers have shown that pregnant adolescents allocate proportionally more choline to maternal brain metabolism compared to adults, potentially at the expense of placental transfer [88].

The current adequate intake for choline during pregnancy is 450 mg/day, as established by the Institute of Medicine [12,89]. However, this recommendation was derived primarily from studies in adult women and does not account for the unique demands of adolescent pregnancy where ongoing maternal growth competes with fetal development. Studies show that pregnant adolescents consume substantially less choline than recommended. Average dietary intake in this population ranges from 250 to 320 mg/day [52,68,98,99], representing only 55–71% of the average intake. Major dietary sources of choline include eggs (147 mg per large egg), beef liver (355 mg per serving), chicken breast (72 mg per serving), salmon (56 mg per serving), soybeans (107 mg per cup), and cruciferous vegetables such as broccoli (31–65 mg per cup) [98,99]. However, adolescents typically avoid organ meats like liver and consume insufficient quantities of eggs and other choline-rich foods. Dairy products (milk provides 38 mg per cup) also contribute to choline intake, but consumption patterns in [68] adolescents are often suboptimal. Table 4 summarizes the key studies on choline status and outcomes in pregnancy. 

Based on metabolic tracer studies and isotope dilution techniques, we can estimate the distribution of choline needs during pregnancy [26,99]:

- Maternal basal metabolism: ~300 mg/day for ongoing cellular functions, including membrane phospholipid turnover, neurotransmitter synthesis (acetylcholine production), and hepatic lipid metabolism (VLDL secretion)
- Adolescent-specific maternal needs: Additional 100–150 mg/day for ongoing brain maturation (myelination of prefrontal cortex and association pathways), skeletal growth, and increased metabolic demands of a still-developing body [100,101,102,103,104,105]
- Placental metabolism: ~50 mg/day for placental tissue growth, phospholipid synthesis for expanding membrane systems, and choline oxidation [106,107]
- Fetal requirements: ~150–200 mg/day during third trimester for rapid brain development (neurogenesis, synaptogenesis, myelination), hepatic maturation, and whole-body growth [12,30,87,106,108]
- Total estimated requirement for pregnant adolescents: 600–700 mg/day

This creates a substantial deficit. With average intake of 280 mg/day and requirements of 650 mg/day, pregnant adolescents face a daily shortfall of approximately 370 mg (57% deficit). This chronic deficiency accumulates throughout pregnancy, potentially compromising both maternal and fetal outcomes. Over a 280-day gestation period, this deficit could exceed 100 g of choline, significantly impacting critical developmental processes [52,68,98,99].

### 4.2. Myelination in Adolescents

While the peak period of myelination occurs in early childhood, significant white matter continues throughout adolescence until 20–25 years old. Longitudinal MRI studies demonstrate that white matter volume increases by 12–15% between ages 15–25, with the most pronounced changes occurring in prefrontal cortex, anterior cingulate cortex, and long-range association fiber tracts connecting frontal, temporal, and parietal regions [100,101,102,103,104,105]. This ongoing myelination is not “residual” development but represents critical maturation of neural circuits subserving executive function, impulse control, and emotional regulation [102,103]. This ongoing myelination requires substantial quantities of phosphatidylcholine, as myelin is approximately 70% lipid by dry weight, with phosphatidylcholine representing 10–15% of total myelin lipids. Given that the human brain contains approximately 500 g of white matter undergoing active maturation during adolescence, this translates to 1–1.5 g of new myelin daily during peak adolescent development periods [100,101,102,103,104,105]. The phosphatidylcholine requirement for this synthesis is estimated at 150–200 mg/day [12,30,100], representing a substantial metabolic demand that directly competes with pregnancy-related choline needs.

The observation that fetal plasma choline concentrations (18–22 μmol/L in third trimester) exceed maternal levels (7–9 μmol/L in pregnant adults, 6–8 μmol/L in pregnant adolescents) appears paradoxical but reflects several important biological mechanisms [106,107]:

- The placenta maintains a concentration gradient through energy-dependent transport mechanisms. CTL1 transporters (SLC44A1) function bidirectionally but show preferential transport from maternal to fetal circulation when coupled with Na^+^-K^+^-ATPase activity and membrane potential gradients [2,6]. This active transport system can concentrate choline against a concentration gradient, like active transport of amino acids and other essential nutrients. The transport capacity is estimated at 0.5–0.8 μmol/min/kg placental tissue, sufficient to maintain elevated fetal levels under normal conditions [2]. The fetal liver has substantially lower choline oxidase and choline dehydrogenase activity (approximately 30–40% of adult levels) compared to maternal liver, resulting in reduced catabolism and higher circulating levels.
- While the fetal brain actively incorporates choline for rapid neurogenesis and membrane synthesis, the overall fetal metabolic rate for choline degradation is 40–50% lower than maternal rates [106]. This metabolic immaturity serves to conserve choline for biosynthetic purposes rather than oxidative metabolism. The fetus actively sequesters choline in developing neural tissues through high-affinity choline transporters (CHT1 and CTL1) expressed at the blood–brain barrier [2,12]. Fetal brain tissue concentrations reach 32–36 μmol/L during the third trimester, representing a 1.5–2-fold concentration gradient over fetal plasma [12]. This preferential accumulation reflects the critical importance of choline for neurodevelopment, with the fetal brain prioritizing choline uptake even when plasma levels are marginal. The placenta itself possesses enzymatic capacity for choline synthesis and metabolism. Placental tissue expresses PEMT (phosphatidylethanolamine N-methyltransferase), which can synthesize phosphatidylcholine de novo, and phospholipases that release free choline from phospholipids [2,109,110]. Placental choline oxidation is minimal (representing less than 5% of choline uptake), preserving choline for transfer to fetal circulation [2]. Additionally, the placenta releases choline metabolites including phosphocholine and glycerophosphocholine into fetal circulation, contributing to elevated total choline-containing compounds.
- Maternal physiology appears to prioritize fetal choline delivery through several adaptive mechanisms, even at the expense of maternal stores [30,31,106]. During pregnancy, upregulation of placental choline transporters increases transfer capacity [37,38]. Maternal choline clearance decreases during pregnancy (from 1.5 mL/min/kg in non-pregnant state to 0.8–1.2 mL/min/kg during pregnancy), conserving choline in circulation for placental uptake [30,31]. Furthermore, maternal liver and skeletal muscle choline concentrations decline progressively during pregnancy while fetal concentrations remain stable or increase, demonstrating net maternal-to-fetal choline flux [30,106,107]. Fetal kidneys exhibit lower choline excretion rates compared to maternal kidneys, with fractional reabsorption exceeding 95% [106]. This efficient renal conservation mechanism contributes to maintaining elevated fetal plasma choline concentrations.

In adolescent pregnancy with suboptimal maternal choline status, this maternal-to-fetal concentration gradient may be compromised. Studies show that when maternal choline status is deficient (plasma levels < 6 μmol/L), fetal concentrations fall proportionally more than maternal levels, suggesting that the active transport mechanisms become saturated or overwhelmed under conditions of substrate limitation [106,107]. Specifically, when maternal plasma choline falls below 5 μmol/L (observed in 15–20% pregnant adolescents with poor dietary intake), the maternal-to-fetal ratio decreases from the normal 2.5:1 to approximately 1.5:1, indicating impaired placental transfer capacity. Furthermore, adolescent pregnancies demonstrate 15–20% reduced placental CTL1 expression compared to adult pregnancies [2,111], potentially limiting the maximal transport capacity even when maternal choline availability is adequate. This combination of reduced maternal supply increased maternal demands (for ongoing adolescent development) and diminished placental transport capacity creates a “triple jeopardy” for fetal choline availability.

### 4.3. Biochemical Consequences of Deficiency

Choline deficiency in adolescent pregnancy triggers a cascade of biochemical alterations. The initial response involves depletion of free choline, followed by mobilization of phosphatidylcholine from cellular membranes through phospholipase D activation [85]. However, adolescents have limited PC reserves, making this compensatory mechanism less effective [112]. As deficiency progresses, several key biochemical markers become evident. Plasma choline concentrations fall below 6 μmol/L (compared to normal pregnancy levels of 8–12 μmol/L), and phosphatidylcholine/sphingomyelin ratios decrease, indicating altered membrane composition [21,113,114]. Additionally, homocysteine levels increase due to impaired betaine-dependent remethylation, suggesting compromised methylation capacity [93].

The impact on neurotransmitter synthesis is particularly significant. Acetylcholine production becomes limiting when choline availability falls below critical thresholds, affecting both maternal cognitive function and fetal cholinergic development [115]. This is evidenced by reduced acetylcholinesterase activity in adolescent pregnancies with low choline status [116].

### 4.4. Developmental Impact on Fetal Brain

The fetal brain is particularly vulnerable to choline deficiency during specific developmental windows. The period of 22–42 weeks post-conception represents a critical phase when neurogenesis, neuronal migration, and synaptogenesis occur at maximal rates [117]. During this period, choline requirements increase dramatically to support membrane synthesis and neurotransmitter production [118].

Choline deficiency during this critical window results in several pathological changes. Neural progenitor cell proliferation is reduced, leading to smaller brain regions, particularly the hippocampus [114]. Cell death through apoptosis is increased, affecting regions involved in memory and learning [119]. Additionally, myelination is impaired, resulting in delayed or abnormal white matter development [120].

At the molecular level, choline deficiency alters gene expression patterns through epigenetic mechanisms. DNA methylation in promoter regions of genes involved in neuroplasticity, such as BDNF and CREB, is reduced, potentially affecting long-term neural function [121,122]. Histone modifications are also altered, creating lasting changes in chromatin structure that persist into adulthood.

**Figure 4:**
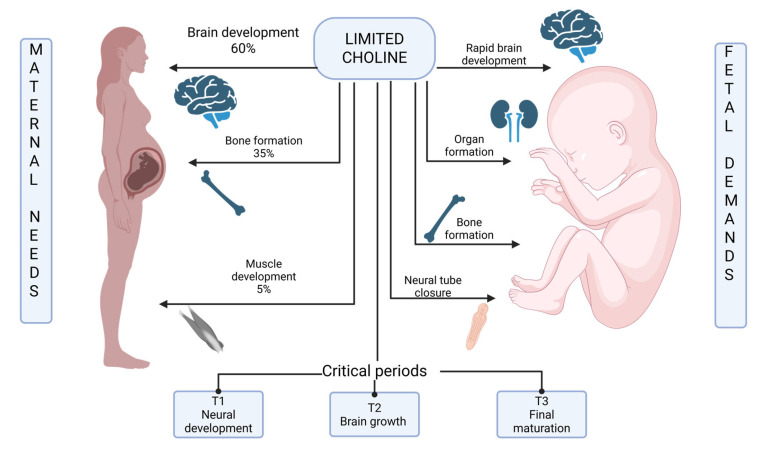
Maternal–Fetal Choline Competition during adolescent pregnancy.

Figure 4 is a comparative diagram showing the allocation of choline resources between maternal needs (ongoing brain development, metabolic demands) and fetal requirements (rapid brain growth, organ development) during adolescent pregnancy.

### 4.5. Systemic Consequences Beyond the Brain

While brain development is the most critical target, choline deficiency in adolescent pregnancy affects multiple organ systems. The liver shows early signs of dysfunction with elevated aminotransferases and reduced very low-density lipoprotein (VLDL) synthesis due to impaired phosphatidylcholine production [123]. This can progress to fatty liver, which is more common in pregnant adolescents with poor nutritional status [124].

Placental function is also compromised by choline deficiency. Reduced phosphatidylcholine synthesis affects membrane integrity and may contribute to the increased risk of placental abruption and preterm birth observed in adolescent pregnancies [125]. Additionally, angiogenesis may be impaired, affecting placental vascularization and nutrient transport capacity [126]. The immune system shows altered function in choline-deficient adolescent pregnancies. Lymphocyte proliferation is reduced, and cytokine production patterns are altered, potentially increasing susceptibility to infections [127]. This immune dysfunction may contribute to the higher rates of chorioamnionitis and other infectious complications in adolescent pregnancies [76,128].

**Table 4:** Summary of Key Studies on Choline Status and Outcomes in Pregnancy.

Author	Study Design	Population	Sample Size (n)	Choline Assessment Method	Main Outcome Measures	Key Findings
Bahnfleth [76]	RCT, double-blind, 7-year follow-up	Pregnant women (18–35 y), subset ages 18–21	n = 140 total (24 young mothers)	Maternal plasma choline measured by LC-MS/MS	Child sustained attention at age 7 years	Supplementation (930 mg/day) improved attention: 15% faster reaction time, 23% fewer lapses (*p* < 0.01)
Jacobson [55]	RCT, double-blind, placebo-controlled	Pregnant women with alcohol exposure (18–35 y)	n = 62 (31 choline, 31 placebo)	Maternal plasma choline at baseline and throughout pregnancy	Infant growth and cognition at 12 months	Choline (2000 mg/day) improved recognition memory (*p* = 0.01) and reduced cognitive errors by 22% (*p* = 0.04)
Wu [14,129]	Prospective cohort	Pregnant women (19–42 y)	n = 154	Maternal plasma free choline and betaine (LC-MS/MS) at 16 weeks	Infant cognitive development at 18 months (Bayley Scales)	Each 1 μmol/L increase in maternal choline associated with 0.11-point higher MDI (*p* = 0.02); 4.8-point difference between tertiles
Boeke [14]	Prospective cohort (Project Viva)	Pregnant women (20–40 y)	n = 1038 mother-child pairs	Validated food frequency questionnaire during pregnancy	Child cognition at age 7 years	Highest choline intake quartile (>449 mg/day) vs. lowest (<237 mg/day): better visual memory
Mellott [130]	Animal model (rat), controlled diet	Pregnant rats	n = 45 (15 per group: supplemented/control/deficient)	Controlled dietary choline: 5.0 g/kg (supplemented), 1.1 g/kg (control), 0 g/kg (deficient); plasma choline	Offspring hippocampal development, MAPK/CREB activation, spatial memory	Supplementation enhanced hippocampal maturation, increased MAPK/CREB phosphorylation (*p* < 0.001), improved memory
Jadavji [131]	Animal model (mouse), controlled diet	Pregnant mice (MTHFR+/+ and MTHFR+/-)	n = 80 (20 per group)	Controlled dietary choline: 0.6 g/kg (deficient) vs. 1.2 g/kg (control); maternal/fetal choline and DNA methylation measured	Offspring hippocampal neurons, apoptosis, memory, DNA methylation	Deficiency: 15–20% fewer neurons (*p* < 0.001), increased apoptosis, impaired memory (*p* < 0.01), altered BDNF/CREB methylation
Wong-Goodrich [132]	Animal model (rat), controlled diet, lifespan study	Pregnant rats with prenatal and adult choline manipulation	n = 64 offspring	Controlled dietary choline: 5 g/kg (supplemented) vs. 1.1 g/kg (control); plasma and brain tissue choline measured	Adult hippocampal plasticity, neurogenesis, spatial memory	Prenatal supplementation: 25% more progenitor cells (*p* < 0.001), enhanced LTP (*p* < 0.01), superior memory performance
Moreno [133]	Animal model (rat), controlled diet	Pregnant rats	n = 40 litters	Controlled dietary choline: 5 g/kg vs. 1.1 g/kg; offspring plasma choline measured	Developmental trajectory of memory function	Supplementation accelerated memory emergence by 3–5 days (*p* < 0.001), enhanced hippocampal-dependent learning
Baumgartner [2]	Human tissue analysis	Placentas from pregnancies (20–40 y)	n = 29 placentas (8–40 weeks gestation)	Placental tissue: CTL1/CTL2 mRNA (qRT-PCR) and protein (Western blot)	Choline transporter expression across gestation	CTL1 increases throughout gestation; localized to syncytiotrophoblast microvillous membrane; peak expression at term
Bernhard [106]	Prospective cohort	Preterm and term infants with mothers	n = 88 mother-infant pairs	Maternal plasma and cord blood choline (LC-MS/MS) at delivery	Maternal–fetal choline concentration gradient	Median cord blood choline (16.3 μmol/L) > maternal plasma (8.9 μmol/L); ratio 1.83:1 (*p* < 0.001); preterm infants lower
Taesuwan [107]	RCT, metabolomics study	Pregnant women (22–35 y)	n = 26 (13 per group)	Comprehensive choline metabolome in maternal plasma, cord blood, placenta (LC-MS/MS)	Choline metabolite profiles across pregnancy and delivery	Supplementation (550 mg/day) increased maternal betaine; altered placental choline partitioning; increased cord blood phosphocholine
Shaw [70]	Case–control study	Women with NTD-affected pregnancies vs. controls	n = 424 cases, 440 controls	Periconceptional dietary choline and betaine intake (FFQ)	Neural tube defects in offspring	Highest quartile choline intake (>498 mg/day) vs. lowest (<290 mg/day): 51% reduced NTD risk (OR 0.49, 95% CI 0.30–0.81)
Shaw [17]	Case–control study	Women with NTD-affected pregnancies vs. controls	n = 330 cases, 680 controls	Maternal plasma total choline, betaine, methionine, vitamers (LC-MS/MS)	Neural tube defects in offspring	Low plasma choline (<5.1 μmol/L) associated with 2.0-fold increased NTD risk (OR 2.0, 95% CI 1.2–3.4), independent of folate
Das [11]	Narrative review	Adolescents (10–19 y)	Review of 89 studies	Systematic literature review	Adolescent nutritional physiology, metabolism, requirements	Adolescents have unique nutritional needs due to ongoing growth; pregnancy compounds demands; many deficiencies documented
Cusick [134]	Narrative review	Prenatal through age 2 years	Review of 127 studies	Systematic literature review	Nutrition and brain development in first 1000 days	Critical periods for nutritional influence on brain development; choline, iron, iodine, folate essential; deficiencies have lasting effects

RCT = Randomized Controlled Trial; FFQ = Food Frequency Questionnaire; LC-MS/MS = Liquid Chromatography-Tandem Mass Spectrometry; qRT-PCR = Quantitative Reverse Transcription Polymerase Chain Reaction; MDI = Mental Development Index (Bayley Scales); LTP = Long-Term Potentiation; MAPK = Mitogen-Activated Protein Kinase; CREB = cAMP Response Element-Binding protein; BDNF = Brain-Derived Neurotrophic Factor; NTD = Neural Tube Defect; OR = Odds Ratio; CI = Confidence Interval; y = years.

## 5. Clinical Implications and Current Evidence

### 5.1. Cognitive and Neurological Outcomes in Offspring

Longitudinal studies examining cognitive outcomes in children born from adolescent mothers have revealed patterns that may be partially attributable to choline deficiency, though early studies did not directly measure maternal choline status.

Early epidemiological studies identified cognitive deficits in offspring of adolescent mothers but did not measure maternal choline status [11,37,135]. These studies found that children of teenage mothers scored 3–5 points lower on standardized cognitive assessments at age 7 years, with deficits most pronounced in memory and attention domains [135]. While these studies did not measure maternal choline status, the specific pattern of cognitive deficits (particularly affecting hippocampus-dependent memory and prefrontal cortex-mediated attention) corresponds to brain regions known to be highly sensitive to prenatal choline availability. These observations generated the hypothesis that nutritional factors, including choline deficiency, might contribute to adverse neurodevelopmental outcomes in offspring of adolescent mothers.

More recent studies specifically examining choline status have provided mechanistic insights and established causal relationships between maternal choline and offspring cognitive outcomes. Bahnfleth et al. [76] conducted a 7-year follow-up of a randomized, double-blind, placebo-controlled trial in pregnant women. Although this study included primarily adult pregnant women (ages 20–35), a subset analysis of younger mothers (ages 18–21, n = 24) demonstrated that prenatal choline supplementation (930 mg/day during third trimester) improved sustained attention in offspring at age 7 years. Maternal plasma choline was directly measured, showing mean concentrations of 12.8 ± 2.1 μmol/L in the supplemented group versus 8.2 ± 1.6 μmol/L in the placebo group (*p* < 0.001). Offspring of supplemented mothers demonstrated 15% faster reaction times and 23% fewer attention lapses on continuous performance tasks [55]. Jacobson et al. conducted a randomized, double-blind, placebo-controlled trial in 62 pregnant women with alcohol exposure. This study measured maternal plasma choline concentrations at baseline and throughout pregnancy, demonstrating mean levels of 7.8 ± 1.9 μmol/L at enrollment. Maternal choline supplementation (2000 mg/day from enrollment through 6.5 months postpartum) significantly improved infant growth and cognitive function at 12 months of age. Infants in the choline group showed better recognition memory (*p* = 0.01) and reduced errors on cognitive tasks (22% improvement, *p* = 0.04) compared to placebo. Wu et al. [129] conducted a prospective cohort study in 154 pregnant women (ages 19–42 years), with direct measurement of maternal plasma choline and betaine in early second trimester (mean 16 weeks gestation). Maternal plasma free choline concentrations ranged from 5.2 to 15.8 μmol/L (mean 8.9 ± 2.3 μmol/L). Higher maternal choline levels were significantly associated with improved infant cognitive development scores at 18 months. Specifically, each 1 μmol/L increase in maternal plasma choline was associated with 0.11-point higher Mental Development Index scores on Bayley Scales (*p* = 0.02) [129]. The relationship was dose-dependent, with infants in the highest tertile of maternal choline (>10.2 μmol/L) scoring 4.8 points higher than those in the lowest tertile (<7.6 μmol/L). Boeke et al. [14] analyzed data from 1038 mother-child pairs in Project Viva cohort. Maternal choline intake was assessed through validated food frequency questionnaires during pregnancy, with mean intake of 315 ± 111 mg/day. Although plasma choline was not measured, dietary choline intake showed significant associations with child cognitive outcomes at age 7 years. Children whose mothers consumed choline in the highest quartile (>449 mg/day) demonstrated better visual memory (*p* = 0.02) compared to the lowest quartile (<237 mg/day) [14]. The association persisted after adjustment for multiple confounders including maternal education, socioeconomic status, and other dietary factors.

Neuroimaging studies have revealed structural brain differences in offspring related to maternal choline status during pregnancy. Complementary animal studies provide mechanistic evidence with precise control of maternal choline status. Mellott et al. [130] used a rat model with controlled dietary choline manipulation. Pregnant rats received either choline-supplemented diet (5.0 g choline chloride/kg diet, approximately 4-fold normal), control diet (1.1 g/kg), or choline-deficient diet (0 g/kg) during gestational days 12–17 (equivalent to second trimester in humans). Maternal plasma choline, fetal plasma choline, and fetal brain choline were all directly measured. Choline-supplemented dams had maternal plasma choline of 45 ± 8 μmol/L, resulting in offspring with enhanced hippocampal development, increased MAPK and CREB activation, and superior spatial memory performance in adulthood. Jadavji et al. [131] demonstrated that pregnant mice fed choline-deficient diets (providing 50% of recommended choline, 0.6 g/kg diet vs. control 1.2 g/kg) during pregnancy produced offspring with 15–20% fewer hippocampal neurons (*p* < 0.001), reduced dendritic branching complexity, and impaired performance in spatial memory tasks (Morris water maze, *p* < 0.01). These effects persisted into adulthood and were associated with altered DNA methylation patterns in genes regulating neuroplasticity (BDNF, CREB, CAMKII). Maternal plasma choline, fetal brain tissue choline, and DNA methylation status were all directly quantified, establishing mechanistic linkages. Wong-Goodrich et al. [132] showed that prenatal choline supplementation (5 g/kg diet) in rats enhanced hippocampal plasticity and memory function in offspring, with effects modulated by adult choline availability. Direct measurements included maternal and offspring plasma choline, brain tissue choline, neurotransmitter levels, and behavioral outcomes. Offspring of supplemented dams demonstrated 25% more hippocampal progenitor cells (*p* < 0.001), enhanced long-term potentiation (*p* < 0.01), and superior spatial memory performance. Moreno & de Brugada [133] demonstrated that prenatal choline supplementation in rats accelerated the emergence of hippocampal-dependent memory function, with quantified measurements of maternal dietary choline intake, offspring plasma choline concentrations, and longitudinal behavioral testing. Supplemented offspring showed memory capabilities 3–5 days earlier in development compared to controls (*p* < 0.001).

The evidence linking choline deficiency to adverse cognitive outcomes in offspring has evolved through multiple lines of investigation. Early observational studies (1980s–2000s) noted cognitive patterns in offspring of adolescent mothers consistent with nutritional deficiencies, but did not measure specific nutrients [11,37,135]. Animal studies (2000–2015) with controlled dietary choline manipulation established causal relationships, demonstrating that prenatal choline deficiency produces specific neurodevelopmental abnormalities [4,12,70,133,136,137,138]. Human observational cohorts with biomarker measurement (2010–2020) correlated maternal choline status with offspring outcomes, establishing dose–response relationships [14,129]. Randomized controlled trials (2018–2022) in humans demonstrated that choline supplementation improves offspring cognitive outcomes, establishing causality [55,76]. Neuroimaging studies (2010-present) linked maternal choline status to structural brain development, providing biological plausibility The convergence of evidence from epidemiological studies, randomized trials, neuroimaging research, and mechanistic animal experiments provides strong support for the critical role of adequate choline status during adolescent pregnancy in optimizing offspring neurodevelopment.

### 5.2. Maternal Health Outcomes

Choline deficiency in adolescent pregnancy is associated with several adverse maternal outcomes. Hepatic dysfunction, manifested as elevated liver enzymes and fatty infiltration, occurs in 15–20% of choline-deficient pregnant adolescents compared to 3–5% in adults [138,139]. This hepatic dysfunction can progress to more serious conditions and may require medical intervention [140].

Cognitive function in adolescent mothers is also affected by choline status. Studies using validated cognitive assessment tools have shown that choline-deficient pregnant adolescents perform more poorly on tests of working memory, attention, and executive function compared to those with adequate choline status [124]. These cognitive deficits may persist in postpartum, potentially affecting parenting capacity and maternal functioning [141,142]. Choline deficiency appears to increase the risk of mood disorders in adolescent mothers. The Edinburgh Postnatal Depression Scale scores are significantly higher in choline-deficient adolescents, and the risk of postpartum depression is increased by 40–50%. This association may reflect the role of choline in neurotransmitter synthesis and mood regulation [143]. Table 5 summarizes the systemic consequences of choline deficiency in adolescent pregnancy. 

### 5.3. Pregnancy Complications and Birth Outcomes

Adolescent pregnancies with inadequate choline status show higher rates of several complications. Neural tube defects occur at a rate of 2.1 per 1000 births in choline-deficient adolescents compared to 0.8 per 1000 in those with adequate status [17]. This association is independent of folate status and appears to reflect the specific role of choline in neural tube closure [74]. Table 6 summarize the metabolic differences of Choline in adolescence and adult pregnant women.

Preterm birth rates are also elevated in choline-deficient adolescent pregnancies (18.5% vs. 12.3% in adequate status), and these infants tend to have lower birth weights and smaller head circumferences [24,147]. The mechanism may involve impaired placental function and reduced nutrient transport capacity [24].

Intrauterine growth restriction (IUGR) shows a strong association with maternal choline status in adolescent pregnancies. The risk of IUGR is increased 2.3-fold when maternal choline concentrations are below the 25th percentile for gestational age [148]. This association is particularly strong for brain growth, with head circumference being disproportionately affected.

**Table 6:** Metabolic Differences: Adolescents vs. Adult Pregnant Women.

Parameter	Adolescents (15–19 years)	Adults (20–35 years)	Clinical Significance	Ref.
Plasma choline (μmol/L)	6.8 ± 1.0	8.5 ± 1.2	Increased deficiency risk	[78,129]
Choline clearance (mL/min/kg)	0.8 ± 0.2	1.2 ± 0.3	Reduced renal handling	[87,149]
Hepatic choline reserves (% of adult)	55 ± 12	100 (reference)	Limited storage capacity	[96,144]
CHKA activity (% increase)	150–200	200–300	Impaired PC synthesis	[3,113,114]
CTL1 expression (% of adult)	80 ± 15	100 (reference)	Reduced placental transport	[3,6]
Methylation capacity (SAM/SAH ratio)	2.8 ± 0.4	3.6 ± 0.5	Compromised epigenetic regulation	[140,150]

### 5.4. Evidence from Animal Models

While human studies provide essential epidemiological and observational data, animal models have been instrumental in elucidating the causal mechanisms by which choline deficiency during pregnancy affects offspring neurodevelopment. These experimental paradigms allow controlled manipulation of maternal choline intake and direct assessment of neurobiological outcomes that cannot be ethically studied in humans.

#### 5.4.1. Rodent Models of Prenatal Choline Supplementation and Deficiency

Most of the mechanistic evidence derives from rat and mouse models, where prenatal choline availability has been experimentally varied across a range from severe deficiency to high supplementation [151,152]. These studies have consistently demonstrated dose-dependent relationships between maternal choline intake and offspring brain development.

Multiple studies using pregnant rat models have shown that prenatal choline supplementation (typically 4–5 times the adequate intake level) produces lasting structural changes in offspring hippocampus. Specifically, choline-supplemented offspring exhibit:

- Increases in hippocampal progenitor cell proliferation during the critical period of neurogenesis (equivalent to human second trimester)
- Enhanced dendritic spine density in CA1 and CA3 pyramidal neurons, with more dendritic spines per unit dendrite length persisting into adulthood
- Larger hippocampal volume maintained throughout the lifespan
- Reduced age-related hippocampal atrophy, suggesting neuroprotective effects extending into senescence
- Conversely, prenatal choline deficiency (typically <25% of adequate intake) produces opposite effects, with smaller hippocampi, reduced neuronal numbers, and impaired neurogenesis.

Neurochemical and Functional Consequences:

- The structural changes induced by prenatal choline availability translate into functional differences in neurotransmitter systems and synaptic plasticity. Offspring of choline-supplemented dams show greater acetylcholine release capacity in hippocampal synapses in response to depolarizing stimuli. This enhanced cholinergic function correlates with improved performance on hippocampus-dependent spatial memory tasks.

#### 5.4.2. Epigenetic Mechanisms

One of the most important insights from animal models concerns the epigenetic mechanisms by which prenatal choline availability programs lasting phenotypic [151]. Choline serves as a methyl donor through its metabolite betaine, influencing DNA and histone methylation patterns during critical developmental windows.

Studies using pregnant rats demonstrate that maternal choline supplementation alters DNA methylation patterns in offspring brain, particularly in genes regulating neuroplasticity and cognitive function. Key findings include altered methylation of the brain-derived neurotrophic factor (BDNF) gene, insulin-like growth factor 2 (IGF2) gene, and cAMP response element-binding protein (CREB) gene. Importantly, these epigenetic modifications are established during prenatal development and persist throughout the lifespan, providing a molecular mechanism for the permanent effects of prenatal choline on cognition. Studies using pregnant mice have shown that these methylation changes can even be transmitted across generations (transgenerational epigenetic inheritance).

#### 5.4.3. Translational Limitations and Considerations

While animal models provide essential mechanistic insights, several important limitations must be considered when translating findings into human adolescent pregnancy:

- Species differences in choline metabolism: Rodents have higher rates of endogenous phosphatidylcholine synthesis via PEMT compared to humans, potentially making them less dependent on dietary choline
- Lack of true adolescent developmental stage: Rodents transition more abruptly from juvenility to reproductive maturity without the extended adolescent growth phase characteristic of humans
- Compressed gestation period: Rat gestation lasts only 21 days versus 40 weeks in humans, compressing developmental events into a much shorter timeframe
- Genetic homogeneity: Inbred laboratory rodent strains lack the genetic diversity of human populations

Despite these limitations, animal models have been essential for establishing causality, elucidating molecular and cellular mechanisms, identifying critical developmental windows, testing dose–response relationships, and examining transgenerational effects.

## 6. Discussion

Current clinical guidelines for choline intake during pregnancy fail to adequately address the unique needs of adolescent mothers. The recommendation of 450 mg/day during pregnancy was established based primarily on studies in adult women and may be insufficient for adolescents [20,125,153,154]. Given the competing demands of ongoing maternal development and fetal growth, adolescent mothers may require 20–30% higher choline intake to maintain adequate status. The challenge in developing adolescent-specific guidelines lies in the limited research conducted specifically in this population. Most studies of choline and pregnancy have excluded or underrepresented adolescents, creating a significant knowledge gap. Additionally, the heterogeneity within the adolescent population (early vs. late adolescence) requires nuanced recommendations based on developmental stage. Professional organizations have not issued specific guidelines for supplementation in adolescent pregnancy, representing a critical gap in clinical care, as healthcare providers lack evidence-based guidance for this vulnerable population.

Developing effective supplementation strategies for pregnant adolescents requires consideration of multiple factors including bioavailability, timing and potential interactions with other nutrients. Choline bitartrate and phosphatidylcholine represent the most used supplemental forms, with phosphatidylcholine showing superior bioavailability but higher cost [54,76,125].

Timing of supplementation appears critical, with the greatest benefits observed when initiated before 16 weeks of gestation [66]. This timing corresponds to the period of rapid neurogenesis and early brain development when choline requirements are highest [124]. But many adolescents present for prenatal care later in pregnancy, limiting the window for optimal intervention. Dosing protocols must consider both safety and efficacy. Studies suggest that adolescent mothers may benefit from 550 to 650 mg/day of choline, representing a 20- 45% increase over current recommendations for adults [20].

Current research on choline and adolescent pregnancy has several important limitations: most studies are observational rather than interventional, limiting causal inferences about the relationship between choline status and outcomes. Randomized controlled trials in pregnant adolescents face ethical and practical challenges, including concerns about withholding potentially beneficial interventions and difficulties with recruitment and retention. The measurement of choline status is complex and may not reflect functional adequacy. Plasma choline concentrations can be influenced by recent dietary intake, time of day, and other factors. Newer biomarkers, including choline metabolites and functional assessments of methylation capacity, may provide better indicators of nutritional status. Third, long-term follow-up studies are challenging to conduct and interpret. Children born to adolescent mothers often face multiple environmental and socioeconomic challenges that can confound the assessment of nutritional effects on development [52]. Distinguishing the specific contributions of choline deficiency from other risk factors requires sophisticated analytical approaches and large sample sizes [62].

Several key research priorities emerge from this review: well-designed intervention studies are needed to establish optimal choline dosing for adolescent pregnancy. These studies should include dose–response relationships, timing of initiation and assessment of both maternal and fetal outcomes. Studies using advanced techniques such as metabolomics and epigenomics could provide insights into the pathways by which choline affects development. Understanding these mechanisms could identify biomarkers for monitoring intervention success and guide personalized approaches to supplementation. Developing effective strategies for delivering choline interventions to adolescent populations, including studies of adherence, acceptability and integration with existing prenatal care systems.

Precision nutrition approaches using genetic and metabolomic data could identify adolescents at highest risk for choline deficiency and guide individualized interventions. Genetic variants affecting choline metabolism, such as polymorphisms in PEMT and MTHFR, could inform supplementation strategies [9,58].

Novel delivery systems for choline supplementation, including sustained-release formulations and combination products, could improve adherence and efficacy. Functional foods enriched with choline and other critical nutrients could provide a more acceptable intervention approach for adolescent populations.

## 7. Conclusions

This narrative review has highlighted the critical importance of choline nutrition in adolescent pregnancy and its profound impact on fetal brain development and long-term cognitive outcomes. The evidence clearly demonstrates that pregnant adolescents represent a unique population with distinct metabolic characteristics, competing nutritional demands, and increased vulnerability to choline deficiency compared to adult pregnant women.

Adolescent mothers exhibit reduced choline clearance, decreased enzyme activities, and diminished placental transport capacity, creating a high-risk scenario for functional choline deficiency; the competition between ongoing maternal neural development and fetal brain growth creates unprecedented demands for choline that cannot be met through current dietary recommendations; choline deficiency during critical developmental windows results in lasting alterations to brain structure and function, with measurable impacts on cognitive performance that persist into childhood and beyond; and current clinical guidelines fail to address the unique needs of adolescent mothers, representing a significant gap in evidence-based care.

Healthcare providers caring for pregnant adolescents should consider routine assessment of choline status and implementation of targeted interventions to prevent deficiency. This may include enhanced dietary counseling focusing on choline-rich foods, consideration of supplementation with doses 20–30% higher than current adult recommendations, and monitoring for signs of deficiency throughout pregnancy.

From a public health perspective, addressing choline deficiency in adolescent pregnancy represents an opportunity for primary prevention of neurodevelopmental disorders with lifelong consequences. The potential for improved cognitive outcomes in offspring, reduced healthcare costs, and enhanced maternal health outcomes justifies investment in research, policy development, and intervention programs targeted to this vulnerable population.

Future research priorities include conducting well-designed intervention trials to establish optimal choline dosing protocols for adolescent pregnancy, developing and validating biomarkers for monitoring choline status and intervention success, investigating the mechanisms by which choline affects fetal brain development using advanced molecular techniques, and implementing population-based strategies for improving choline nutrition in adolescent mothers.

The evidence presented in this review addresses choline deficiency in adolescent pregnancy through research, clinical practice changes, and policy initiatives. The window of opportunity for optimizing fetal brain development is narrow, and interventions must be implemented early in pregnancy to achieve maximum benefit. By recognizing and addressing the unique nutritional needs of pregnant adolescents, we can improve outcomes for both mothers and their children, with benefits extending across generations.

## Figures and Tables

**Table 2:** Key Enzymes in Choline Metabolism During Pregnancy.

Enzyme	Function	Adult Activity (% Change)	Adolescent Activity (% Change)	Km (μM)	Ref.
Choline Kinase α	PC synthesis	+200–300%	+150–200%	75–100	[76,77]
CTP:PC Cytidylyltransferase	PC synthesis	+150–200%	+80–120%	25–40	[78,79]
Choline Acetyltransferase	ACh synthesis	+120–150%	+80–100%	300–450	[30,80,81]
Choline Dehydrogenase	Betaine synthesis	+50–75%	+100–125%	150–250	[30,82]
PEMT	Alternative PC synthesis	+100–150%	+50–80%	-	[83,84]

**Table 3:** Placental Choline Transporters—Functional Characteristics.

Transporter	Gene	Protein (kDa)	Choline (μM)	Vmax (Relative)	Placental Localization	Adolescent Expression	References
CTL1	SLC44A1	70	5–20	100% (reference)	Microvillous membrane	80–85% of adult	[86]
CTL2	SLC44A2	68	50–100	30–40%	Both membranes	85–90% of adult	[6]
OCT1	SLC22A1	61	100–500	15–20%	Basal membrane	90–95% of adult	[87]
OCT2	SLC22A2	62	200–400	10–15%	Maternal endothelium	95–100% of adult	[87,88]
OCT3	SLC22A3	58	30–80	25–30%	Syncytiotrophoblast	75–80% of adult	[12,89]

**Table 5:** Systemic Consequences of Choline Deficiency in Adolescent Pregnancy.

System	Pathophysiological Mechanism	Clinical Manifestations	Reversibility	Ref.
Nervous System	Reduced PC synthesis, impaired ACh production, altered methylation	Cognitive deficits, NTDs, reduced memory function	Partially reversible	[22,127]
Hepatic System	Decreased VLDL synthesis, triglyceride accumulation	Fatty liver, elevated transaminases	Reversible	[144]
Cardiovascular	Endothelial dysfunction, altered lipid metabolism	Hypertension, preeclampsia risk	Partially reversible	[145]
Immune System	Altered lymphocyte function, cytokine dysregulation	Increased infection risk, inflammatory complications	Reversible	[146]
Placental Function	Membrane instability, impaired angiogenesis	IUGR, preterm birth, abruption	Partially reversible	[26,62,74]

## Abbreviations

The following abbreviations are used in this manuscript:

PC	Phosphatidylcholine
CK	Choline Kinase
CHKA	Choline Kinase α (Alpha)
CCT	Phosphocholine Cytidylyltransferase
ACh	Acetylcholine
ChAT	Choline acetyltransferase
SAM	S-adenosylmethionine
SAH	S-adenosylhomocysteine
PEMT	Phosphatidylethanolamine N-methyltransferase
VLDL	Very Low-Density Lipoprotein
SM	Sphingomyelin
CTL1	Choline Transporter-Like Protein 1 (SLC44A1)
CTL2	Choline Transporter-Like Protein 2 (SLC44A2)
OCT1	Organic Cation Transporter 1 (SLC22A1)
OCT2	Organic Cation Transporter 2 (SLC22A2)
OCT3	Organic Cation Transporter 3 (SLC22A3)
Km	Michaelis-Menten Constant
Vmax	Maximum Enzymatic Velocity
BDNF	Brain-Derived Neurotrophic Factor
CREB	cAMP Response Element-Binding Protein
NTDs	Neural Tube Defects
IUGR	Intrauterine Growth Restriction

## References

[1] W.H.O. (2007). Adolescent Pregnancy: Unmet Needs and Undone Deeds.

[2] Baumgartner H.K., Trinder K.M., Galimanis C.E., Post A., Wilcox J., Dawson P.A. (2015). Characterization of choline transporters in the human placenta over gestation. Placenta.

[3] Michel V., Yuan Z., Ramsubir S., Bakovic M. (2006). Choline transport for phospholipid synthesis. Exp. Biol. Med..

[4] Blusztajn J.K., Mellott T.J. (2012). Choline nutrition programs brain development via DNA and histone methylation. Cent. Nerv. Syst. Agents Med. Chem..

[5] Medicine I. (1998). Dietary Reference Intakes for Thiamin, Riboflavin, Niacin, Vitamin B6, Folate, Vitamin B12, Pantothenic Acid, Biotin, and Choline.

[6] Hedtke V., Bakovic M. (2019). Choline transport for phospholipid synthesis: An emerging role of choline transporter-like protein 1. Exp. Biol. Med..

[7] Ridgway N.D. (2013). The role of phosphatidylcholine and choline metabolites to cell proliferation and survival. Crit. Rev. Biochem. Mol. Biol..

[8] Paoletti L., Elena C., Domizi P., Banchio C. (2011). Role of phosphatidylcholine during neuronal differentiation. IUBMB Life.

[9] Yuan Z., Tie A., Tarnopolsky M., Bakovic M. (2006). Genomic organization, promoter activity, and expression of the human choline transporter-like protein 1. Physiol. Genom..

[10] Marvin-Dowle K., Burley V.J., Soltani H. (2016). Nutrient intakes and nutritional biomarkers in pregnant adolescents: A systematic review of studies in developed countries. BMC Pregnancy Childbirth.

[11] Das J.K., Salam R.A., Thornburg K.L., Prentice A.M., Campisi S., Lassi Z.S., Koletzko B., Bhutta Z.A. (2017). Nutrition in adolescents: Physiology, metabolism, and nutritional needs. Ann. N. Y. Acad. Sci..

[12] Zeisel S.H. (2006). Choline: Critical role during fetal development and dietary requirements in adults. Annu. Rev. Nutr..

[13] Innis S.M. (2008). Dietary omega 3 fatty acids and the developing brain. Brain Res..

[14] Boeke C.E., Gillman M.W., Hughes M.D., Rifas-Shiman S.L., Villamor E., Oken E. (2013). Choline intake during pregnancy and child cognition at age 7 years. Am. J. Epidemiol..

[15] Veena S.R., Gale C.R., Krishnaveni G.V., Kehoe S.H., Srinivasan K., Fall C.H. (2016). Association between maternal nutritional status in pregnancy and offspring cognitive function during childhood and adolescence; a systematic review. BMC Pregnancy Childbirth.

[16] Caudill M.A. (2010). Pre- and postnatal health: Evidence of increased choline needs. J. Am. Diet. Assoc..

[17] Shaw G.M., Carmichael S.L., Yang W., Selvin S., Schaffer D.M. (2004). Periconceptional dietary intake of choline and betaine and neural tube defects in offspring. Am. J. Epidemiol..

[18] Fisher M.C., Zeisel S.H., Mar M.H., Sadler T.W. (2001). Inhibitors of choline uptake and metabolism cause developmental abnormalities in neurulating mouse embryos. Teratology.

[19] Hochberg Z., Belsky J. (2013). Evo-devo of human adolescence: Beyond disease models of early puberty. BMC Med..

[20] Wallace T.C., Blusztajn J.K., Caudill M.A., Klatt K.C., Natker E., Zeisel S.H., Zelman K.M. (2020). Choline: The neurocognitive essential nutrient of interest to obstetricians and gynecologists. J. Diet. Suppl..

[21] Craciunescu C.N., Albright C.D., Mar M.H., Song J., Zeisel S. (2003). Choline availability during embryonic development alters progenitor cell mitosis in developing mouse hippocampus. J. Nutr..

[22] Meck W.H., Williams C.L. (2003). Metabolic imprinting of choline by its availability during gestation: Implications for memory and attentional processing across the lifespan. Neurosci. Biobehav. Rev..

[23] Niculescu M.D., Craciunescu C.N., Zeisel S.H. (2006). Dietary choline deficiency alters global and gene-specific DNA methylation in the developing hippocampus of mouse fetal brains. FASEB J..

[24] Fullerton M.D., Wagner L., Yuan Z., Bakovic M. (2006). Impaired trafficking of choline transporter-like protein-1 at plasma membrane and inhibition of choline transport in THP-1 monocyte-derived macrophages. Am. J. Physiol. Cell Physiol..

[25] Marcucci H., Paoletti L., Jackowski S., Banchio C. (2010). Phosphatidylcholine biosynthesis during neuronal differentiation and its role in cell fate determination. J. Biol. Chem..

[26] Kwan S.T.C., King J.H., Yan J., Wang Z., Jiang X., Hutzler J.S., Klein H.R., Brenna J.T., Roberson M.S., Caudill M.A. (2017). Maternal choline supplementation modulates placental nutrient transport and metabolism in late gestation of mouse pregnancy. J. Nutr..

[27] Lewis E.D., Richard C., Larsen B.M., Field C.J. (2017). The importance of human milk for immunity in preterm infants. Clin. Perinatol..

[28] Larqué E., Gil-Sánchez A., Prieto-Sánchez M.T., Koletzko B. (2012). Omega 3 fatty acids, gestation and pregnancy outcomes. Br. J. Nutr..

[29] Bernardi J.R., Escobar R.S., Ferreira C.F., Silveira P.P. (2012). Fetal and neonatal levels of omega-3: Effects on neurodevelopment, nutrition, and growth. Sci. World J..

[30] Yan J., Jiang X., West A.A., Perry C.A., Malysheva O.V., Devapatla S., Pressman E., Vermeylen F., Caudill M.A. (2012). Maternal choline intake modulates maternal and fetal biomarkers of choline metabolism in humans. Am. J. Clin. Nutr..

[31] Zeisel S.H. (2007). Gene response elements, genetic polymorphisms and epigenetics influence the human dietary requirement for choline. IUBMB Life.

[32] Sherzai D., Moness R., Sherzai S., Sherzai A. (2023). A systematic review of omega-3 fatty acid consumption and cognitive outcomes in neurodevelopment. Am. J. Lifestyle Med..

[33] Hacker A.N., Fung E.B., King J.C. (2012). Role of calcium during pregnancy: Maternal and fetal needs. Nutr. Rev..

[34] Golden N.H., Nutrition A.S.C. (2014). on: Optimizing bone health in children and adolescents. Pediatrics.

[35] Kovacs C.S. (2001). Calcium and bone metabolism in pregnancy and lactation. J. Clin. Endocrinol. Metab..

[36] Moran V.H. (2007). Nutritional status in pregnant adolescents: A systematic review of biochemical markers. Matern. Child Nutr..

[37] Nupo S.S., Martínez-De la Fuente V., Cruz G.O., Cortes-Hernandez J.L. (2024). Teenage pregnancy and micronutrient deficiency: A critical review. Tecnociencia Chihuah..

[38] Rosenberg K., McEwan H.P. (1991). Teenage pregnancy in Scotland: Trends and risks. Scott. Med. J..

[39] Williamson C.S. (2006). Nutrition in pregnancy. Nutr. Bull..

[40] Chantry C.J., Auinger P., Byrd R.S. (2004). Lactation among adolescent mothers and subsequent bone mineral density. Arch. Pediatr. Adolesc. Med..

[41] Behere R.V., Deshmukh A.S., Otiv S., Gupte M.D., Yajnik C.S. (2021). Maternal vitamin B12 status during pregnancy and its association with outcomes of pregnancy and health of the offspring: A systematic review and implications for policy in India. Front. Endocrinol..

[42] Mamabolo R.L., Alberts M., Steyn N.P., Levitt N.S. (2006). The effect of maternal glucose metabolism, iron, vitamin B12 and folate status on pregnancy outcomes. S. Afr. J. Clin. Nutr..

[43] Wellinghausen N. (2001). Immunobiology of gestational zinc deficiency. Br. J. Nutr..

[44] de Moraes M.L., Barbosa R.d.F., Santo R.E., Santos F.d.S., de Jesus E.F.O., Sardinha F.L.d.C., Carmo M.d.G.T.D. (2011). Maternal-fetal distribution of calcium, iron, copper, and zinc in pregnant teenagers and adults. Biol. Trace Elem. Res..

[45] Swanson C.A., King J.C. (1987). Zinc and pregnancy outcome. Am. J. Clin. Nutr..

[46] Deshpande J.D., Joshi M.M., Giri P.A. (2013). Zinc: The trace element of major importance in human nutrition and health. Int. J. Med. Sci. Public Health.

[47] Woo Q.Y., Lee B.T.K., Lim L.W., Zhang J., Tashiro A., Lau P.K., Thibault G., Wang Y., Lin V.C.L. (2025). Choline intake during pregnancy influences maternal cognitive function and hippocampal gene expression in late adulthood. Nutr. Neurosci..

[48] Ray J.G., Wyatt P.R., Thompson M.D., Vermeulen M.J., Meier C., Wong P.-Y., Farrell S.A., Cole D.E.C. (2007). Vitamin B12 and the risk of neural tube defects in a folic-acid-fortified population. Epidemiology.

[49] Molloy A.M., Kirke P.N., Troendle J.F., Burke H., Sutton M., Brody L.C., Scott J.M., Mills J.L. (2009). Maternal vitamin B12 status and risk of neural tube defects in a population with high neural tube defect prevalence and no folic Acid fortification. Pediatrics.

[50] Finkelstein J.L., Layden A.J., Stover P.J. (2015). Vitamin B-12 and perinatal health. Adv. Nutr..

[51] Derbyshire E., Obeid R., Schön C. (2021). Habitual choline intakes across the childbearing years: A review. Nutrients.

[52] Nguyen H.T., Oktayani P.P.I., Lee S.-D., Huang L.-C. (2025). Choline in pregnant women: A systematic review and meta-analysis. Nutr. Rev..

[53] Spoelstra S.K., Eijsink J.J.H., Hoenders H.J.R., Knegtering H. (2023). Maternal choline supplementation during pregnancy to promote mental health in offspring. Early Interv. Psychiatry.

[54] Caudill M.A., Strupp B.J., Muscalu L., Nevins J.E.H., Canfield R.L. (2018). Maternal choline supplementation during the third trimester of pregnancy improves infant information processing speed: A randomized, double-blind, controlled feeding study. FASEB J..

[55] Jacobson S.W., Carter R.C., Molteno C.D., Stanton M.E., Herbert J.S., Lindinger N.M., Lewis C.E., Dodge N.C., Hoyme H.E., Zeisel S.H. (2018). Efficacy of maternal choline supplementation during pregnancy in mitigating adverse effects of prenatal alcohol exposure on growth and cognitive function: A randomized, double-blind, placebo-controlled clinical trial. Alcohol. Clin. Exp. Res..

[56] Ross R.G., Hunter S.K., McCarthy L., Beuler J., Hutchison A.K., Wagner B.D., Leonard S., Stevens K.E., Freedman R. (2013). Perinatal choline effects on neonatal pathophysiology related to later schizophrenia risk. Am. J. Psychiatry.

[57] Mudd A.T., Getty C.M., Sutton B.P., Dilger R.N. (2016). Perinatal choline deficiency delays brain development and alters metabolite concentrations in the young pig. Nutr. Neurosci..

[58] Ganz A.B., Klatt K.C., Caudill M.A. (2017). Common genetic variants alter metabolism and influence dietary choline requirements. Nutrients.

[59] Korsmo H.W., Jiang X., Caudill M.A. (2019). Choline: Exploring the growing science on its benefits for moms and babies. Nutrients.

[60] Rubinchik-Stern M., Shmuel M., Bar J., Kovo M., Eyal S. (2018). Adverse placental effects of valproic acid: Studies in perfused human placentas. Epilepsia.

[61] Taylor A. (2019). SLC44A1 Transport of Choline and Ethanolamine in Disease. Ph.D. Thesis.

[62] Zeisel S.H., Klatt K.C., Caudill M.A. (2018). Choline. Adv. Nutr..

[63] Wallace T.C., Fulgoni V.L. (2016). Assessment of total choline intakes in the United States. J. Am. Coll. Nutr..

[64] Chester D.N., Goldman J.D., Ahuja J.K., Moshfegh A.J. (2007). Dietary intakes of choline: What we eat in America, NHANES 2007–2008. Food Surveys Research Group Dietary Data Brief.

[65] Bailey R.L., Pac S.G., Fulgoni V.L., Reidy K.C., Catalano P.M. (2019). Estimation of total usual dietary intakes of pregnant women in the United States. JAMA Netw. Open.

[66] Klatt K.C., McDougall M.Q., Malysheva O.V., Taesuwan S., Loinard-González A.A.P., Nevins J.E.H., Beckman K., Bhawal R., Anderson E., Zhang S. (2022). Prenatal choline supplementation improves biomarkers of maternal docosahexaenoic acid status among pregnant participants consuming supplemental DHA: A randomized controlled trial. Am. J. Clin. Nutr..

[67] Wiedeman A.M., Barr S.I., Green T.J., Xu Z., Innis S.M., Kitts D.D. (2018). Dietary choline intake: Current state of knowledge across the life cycle. Nutrients.

[68] Lewis E.D., Subhan F.B., Bell R.C., McCargar L.J., Curtis J.M., Jacobs R.L., Field C.J., APrON team (2014). Estimation of choline intake from 24 h dietary intake recalls and contribution of egg and milk consumption to intake among pregnant and breastfeeding women in Alberta. Br. J. Nutr..

[69] Masih S.P., Plumptre L., Ly A., Berger H., Lausman A.Y., Croxford R., Kim Y.-I., O’cOnnor D.L. (2015). Pregnant Canadian women achieve recommended intakes of one-carbon nutrients through prenatal supplementation but the supplement composition, including choline, requires reconsideration. J. Nutr..

[70] Carmichael S.L., Yang W., Shaw G.M. (2010). Periconceptional nutrient intakes and risks of neural tube defects in California. Birth Defects Res. A Clin. Mol. Teratol..

[71] Poly C., Massaro J.M., Seshadri S., Wolf P.A., Cho E., Krall E., Jacques P.F., Au R. (2011). The relation of dietary choline to cognitive performance and white-matter hyperintensity in the Framingham Offspring Cohort. Am. J. Clin. Nutr..

[72] Bidulescu A., Chambless L.E., Siega-Riz A.M., Zeisel S.H., Heiss G. (2007). Usual choline and betaine dietary intake and incident coronary heart disease: The Atherosclerosis Risk in Communities (ARIC) study. BMC Cardiovasc. Disord..

[73] Zheng Y., Li Y., Rimm E.B., Hu F.B., Albert C.M., Rexrode K.M., Manson J.E., Qi L. (2016). Dietary phosphatidylcholine and risk of all-cause and cardiovascular-specific mortality among US women and men. Am. J. Clin. Nutr..

[74] Mills J.L., Fan R., Brody L.C., Liu A., Ueland P.M., Wang Y., Kirke P.N., Shane B., Molloy A.M. (2014). Maternal choline concentrations during pregnancy and choline-related genetic variants as risk factors for neural tube defects. Am. J. Clin. Nutr..

[75] Obeid R., Derbyshire E., Schön C. (2022). Association between maternal choline, fetal brain development, and child neurocognition: Systematic review and meta-analysis of human studies. Adv. Nutr..

[76] Bahnfleth C.L., Strupp B.J., Caudill M.A., Canfield R.L. (2022). Prenatal choline supplementation improves child color-word inhibition performance aged 7 years in a randomized trial. J. Nutr..

[77] Freedman R., Hunter S.K., Law A.J., Wagner B.D., D’Alessandro A., Christians U., Noonan K., Wyrwa A., Hoffman M.C. (2019). Higher gestational choline levels in maternal infection are protective for infant brain development. J. Pediatr..

[78] Velzing-Aarts F.V., Holm P.I., Fokkema M.R., van der Dijs F.P., Ueland P.M., Muskiet F.A. (2005). Plasma choline and betaine and their relation to plasma homocysteine in normal pregnancy. Am. J. Clin. Nutr..

[79] Ilcol Y.O., Ozbek R., Hamurtekin E., Ulus I.H. (2005). Choline status in newborns, infants, children, breast-feeding women, breast-fed infants and human breast milk. J. Nutr. Biochem..

[80] Holmes-McNary M.Q., Cheng W.L., Mar M.H., Fussell S., Zeisel S.H. (1996). Choline and choline esters in human and rat milk and in infant formulas. Am. J. Clin. Nutr..

[81] Davenport C., Yan J., Taesuwan S., Shields K., West A.A., Jiang X., Perry C.A., Malysheva O.V., Stabler S.P., Allen R.H. (2015). Choline intakes exceeding recommendations during human lactation improve breast milk choline content by increasing PEMT pathway metabolites. J. Nutr. Biochem..

[82] Stammers A.-L., Lowe N.M., Medina M.W., Patel S., Dykes F., Pérez-Rodrigo C., Serra-Majam L., Nissensohn M., Moran V.H. (2015). The relationship between zinc intake and growth in children aged 1-8 years: A systematic review and meta-analysis. Eur. J. Clin. Nutr..

[83] Leung B.M.Y., Kaplan B.J. (2009). Perinatal depression: Prevalence, risks, and the nutrition link—A review of the literature. J. Am. Diet. Assoc..

[84] Christian P., Murray-Kolb L.E., Khatry S.K., Katz J., Schaefer B.A., Cole P.M., LeClerq S.C., Tielsch J.M. (2010). Prenatal micronutrient supplementation and intellectual and motor function in early school-aged children in Nepal. JAMA.

[85] Sweiry J.H., Page K.R., Dacke C.G., Abramovich D.R., Yudilevich D.L. (1986). Evidence of saturable uptake mechanisms at maternal and fetal sides of the perfused human placenta by rapid paired-tracer dilution: Studies with calcium and choline. J. Dev. Physiol..

[86] Traiffort E., O’Regan S., Ruat M. (2013). The choline transporter-like family SLC44: Properties and roles in human diseases. Mol. Asp. Med..

[87] Sanders L.M., Zeisel S.H. (2007). Choline: Dietary requirements and role in brain development. Nutr. Today.

[88] West A.A., Yan J., Jiang X., Perry C.A., Innis S.M., Caudill M.A. (2013). Choline intake influences phosphatidylcholine DHA enrichment in nonpregnant women but not in pregnant women in the third trimester. Am. J. Clin. Nutr..

[89] EFSA Panel on Dietetic Products, Nutrition and Allergies (NDA) (2016). Dietary reference values for choline. EFSA J..

[90] Casey B.J., Jones R.M., Somerville L.H. (2011). Braking and accelerating of the adolescent brain. J. Res. Adolesc..

[91] Hayward C.E., Greenwood S.L., Sibley C.P., Baker P.N., Jones R.L. (2012). Effect of maternal age and growth on placental nutrient transport: Potential mechanisms for teenagers’ predisposition to small-for-gestational-age birth?. Am. J. Physiol.-Endocrinol. Metab..

[92] Zhu X.Z., Deng Z.M., Dai F.F., Liu H., Cheng Y.X. (2023). The impact of early pregnancy metabolic disorders on pregnancy outcome and the specific mechanism. Eur. J. Med. Res..

[93] Black M.M. (2008). Effects of vitamin B12 and folate deficiency on brain development in children. Food Nutr. Bull..

[94] Rush D. (2000). Nutrition and maternal mortality in the developing world. Am. J. Clin. Nutr..

[95] Allen L.H. (2005). Multiple micronutrients in pregnancy and lactation: An overview. Am. J. Clin. Nutr..

[96] Yaworski R. (2017). The Regulation of Hepatic Choline Transport. Master’s Thesis.

[97] Craciunescu C.N., Brown E.C., Mar M.-H., Albright C.D., Nadeau M.R., Zeisel S.H. (2004). Folic acid deficiency during late gestation decreases progenitor cell proliferation and increases apoptosis in fetal mouse brain. J. Nutr..

[98] Zeisel S.H. (2013). Nutrition in pregnancy: The argument for including a source of choline. Int. J. Womens Health.

[99] Christifano D.N., Chollet-Hinton L., Hoyer D., Schmidt K.A., Wasser H.M., McRitchie S.L., Sumner S., Newby P.K., Thompson A.L., Siega-Riz A.M. (2023). Intake of eggs, choline, lutein, zeaxanthin, and DHA during pregnancy and their relationship to fetal neurodevelopment. Nutr. Neurosci..

[100] Barnea-Goraly N., Menon V., Eckert M., Tamm L. (2005). White matter development during childhood and adolescence: A cross-sectional diffusion tensor imaging study. Cereb. Cortex.

[101] Nagy Z., Westerberg H., Klingberg T. (2004). Maturation of white matter is associated with the development of cognitive functions during childhood. J. Cogn. Neurosci..

[102] Sousa S.S., Amaro E., Crego A., Gonçalves Ó.F., Sampaio A. (2018). Developmental trajectory of the prefrontal cortex: A systematic review of diffusion tensor imaging studies. Brain Imaging Behav..

[103] Shaw G.A., Dupree J.L., Neigh G.N. (2020). Adolescent maturation of the prefrontal cortex: Role of stress and sex in shaping adult risk for compromise. Genes Brain Behav..

[104] Vanes L.D., Moutoussis M., Ziegler G. (2020). White matter tract myelin maturation and its association with general psychopathology in adolescence and early adulthood. Hum. Brain Mapp..

[105] Klingberg T., Vaidya C.J., Gabrieli J.D.E., Moseley M.E., Hedehus M. (1999). Myelination and organization of the frontal white matter in children: A diffusion tensor MRI study. Neuroreport.

[106] Bernhard W., Raith M., Kunze R., Koch V. (2015). Choline concentrations are lower in postnatal plasma of preterm infants than in cord plasma. Eur. J. Nutr..

[107] Taesuwan S., McDougall M.Q., Malysheva O.V., Bender E. (2021). Choline metabolome response to prenatal choline supplementation across pregnancy: A randomized controlled trial. FASEB J..

[108] Zeisel S.H., Niculescu M.D. (2006). Perinatal choline influences brain structure and function. Nutr. Rev..

[109] Zeisel S.H. (2006). The fetal origins of memory: The role of dietary choline in optimal brain development. J. Pediatr..

[110] Chmurzynska A., Seremak-Mrozikiewicz A., Malinowska A.M. (2020). Associations between folate and choline intake, homocysteine metabolism, and genetic polymorphism of MTHFR, BHMT and PEMT in healthy pregnant Polish women. Nutr. Diet..

[111] Michel V., Bakovic M. (2012). The ubiquitous choline transporter SLC44A1. Cent. Nerv. Syst. Agents Med. Chem..

[112] Garner S.C., Mar M.H., Zeisel S.H. (1995). Choline distribution and metabolism in pregnant rats and fetuses are influenced by the choline content of the maternal diet. J. Nutr..

[113] Albright C.D., Friedrich C.B., Brown E.C., Mar M.-H., Zeisel S.H. (1999). Maternal dietary choline availability alters mitosis, apoptosis and the localization of TOAD-64 protein in the developing fetal rat septum. Brain Res. Dev. Brain Res..

[114] Napoli I., Blusztajn J.K., Mellott T.J. (2008). Prenatal choline supplementation in rats increases the expression of IGF2 and its receptor IGF2R and enhances IGF2-induced acetylcholine release in hippocampus and frontal cortex. Brain Res..

[115] Moon J., Chen M., Gandhy S.U., Strawderman M., Levitsky D.A., Maclean K.N., Strupp B.J. (2010). Perinatal choline supplementation improves cognitive functioning and emotion regulation in the Ts65Dn mouse model of Down syndrome. Behav. Neurosci..

[116] Thomas J.D., Garrison M., O’Neill T.M. (2004). Perinatal choline supplementation attenuates behavioral alterations associated with neonatal alcohol exposure in rats. Neurotoxicology Teratol..

[117] Ryan S.H., Williams J.K., Thomas J.D. (2008). Choline supplementation attenuates learning deficits associated with neonatal alcohol exposure in the rat: Effects of varying the timing of choline administration. Brain Res..

[118] Glenn M.J., Gibson E.M., Kirby E.D., Mellott T.J., Blusztajn J.K., Williams C.L. (2007). Prenatal choline availability modulates hippocampal neurogenesis and neurogenic responses to enriching experiences in adult female rats. Eur. J. Neurosci..

[119] Holler T., Cermak J.M., Blusztajn J.K. (1996). Dietary choline supplementation in pregnant rats increases hippocampal phospholipase D activity of the offspring. FASEB J..

[120] Blusztajn J.K., Cermak J.M., Holler T., Jackson D.A. (1998). Imprinting of hippocampal metabolism of choline by its availability during gestation: Implications for cholinergic neurotransmission. J. Physiol..

[121] Cermak J.M., Holler T., Jackson D.A., Blusztajn J.K. (1998). Prenatal availability of choline modifies development of the hippocampal cholinergic system. FASEB J..

[122] Pyapali G.K., Turner D.A., Williams C.L., Meck W.H., Swartzwelder H.S. (1998). Prenatal dietary choline supplementation decreases the threshold for induction of long-term potentiation in young adult rats. J. Neurophysiol..

[123] Montoya D.A., White A.M., Williams C.L., Blusztajn J.K., Meck W.H., Swartzwelder H. (2000). Prenatal choline exposure alters hippocampal responsiveness to cholinergic stimulation in adulthood. Brain Res. Dev. Brain Res..

[124] Williams C.L., Meck W.H., Heyer D.D., Loy R. (1998). Hypertrophy of basal forebrain neurons and enhanced visuospatial memory in perinatally choline-supplemented rats. Brain Res..

[125] Meck W.H., Smith R.A., Williams C.L. (1988). Pre- and postnatal choline supplementation produces long-term facilitation of spatial memory. Dev. Psychobiol..

[126] McCann J.C., Hudes M., Ames B.N. (2006). An overview of evidence for a causal relationship between dietary availability of choline during development and cognitive function in offspring. Neurosci. Biobehav. Rev..

[127] Zhu X., Mar M.H., Song J., Zeisel S.H. (2004). Deletion of the Pemt gene increases progenitor cell mitosis, DNA and protein methylation and decreases calretinin expression in embryonic day 17 mouse hippocampus. Brain Res. Dev. Brain Res..

[128] Niculescu M.D., Yamamuro Y., Zeisel S.H. (2004). Choline availability modulates human neuroblastoma cell proliferation and alters the methylation of the promoter region of the cyclin-dependent kinase inhibitor 3 gene. J. Neurochem..

[129] Wu B.T.F., Dyer R.A., King D.J., Richardson K.J., Innis S.M. (2012). Early second trimester maternal plasma choline and betaine are related to measures of early cognitive development in term infants. PLoS ONE.

[130] Mellott T.J., Williams C.L., Meck W.H., Blusztajn J.K. (2004). Prenatal choline supplementation advances hippocampal development and enhances MAPK and CREB activation. FASEB J..

[131] Jadavji N.M., Deng L., Malysheva O., Caudill M.A., Rozen R. (2015). MTHFR deficiency or reduced intake of folate or choline in pregnant mice results in impaired short-term memory and increased apoptosis in the hippocampus of wild-type offspring. Neuroscience.

[132] Wong-Goodrich S.J.E., Glenn M.J., Mellott T.J., Blusztajn J.K. (2008). Spatial memory and hippocampal plasticity are differentially sensitive to the availability of choline in adulthood as a function of choline supply in utero. Brain Res..

[133] Moreno H., de Brugada I. (2021). Prenatal dietary choline supplementation modulates long-term memory development in rat offspring. Nutr. Neurosci..

[134] Cusick S.E., Georgieff M.K. (2016). The role of nutrition in brain development: The golden opportunity of the “first 1000 days". J. Pediatr..

[135] Prado E.L., Dewey K.G. (2014). Nutrition and brain development in early life. Nutr. Rev..

[136] Zeisel S.H. (2000). Choline: Needed for normal development of memory. J. Am. Coll. Nutr..

[137] Sandstrom N.J., Loy R., Williams C.L. (2002). Prenatal choline supplementation increases NGF levels in the hippocampus and frontal cortex of young and adult rats. Brain Res..

[138] Resseguie M.E., da Costa K.-A., Galanko J.A., Patel M., Davis I.J., Zeisel S.H. (2011). Aberrant estrogen regulation of PEMT results in choline deficiency-associated liver dysfunction. J. Biol. Chem..

[139] Corbin K.D., Zeisel S.H. (2012). Choline metabolism provides novel insights into nonalcoholic fatty liver disease and its progression. Curr. Opin. Gastroenterol..

[140] Davison J.M., Mellott T.J., Kovacheva V.P., Blusztajn J.K. (2009). Gestational choline supply regulates methylation of histone H3, expression of histone methyltransferases G9a (Kmt1c) and Suv39h1 (Kmt1a), and DNA methylation of their genes in rat fetal liver and brain. J. Biol. Chem..

[141] Imdad A., Lassi Z., Salaam R., Bhutta Z.A. (2017). Prenatal nutrition and nutrition in pregnancy: Effects on long-term growth and development. Early Nutrition and Long-Term Health.

[142] Irvine N., England-Mason G., Field C.J., Dewey D., Aghajafari F. (2022). Prenatal folate and choline levels and brain and cognitive development in children: A critical narrative review. Nutrients.

[143] Morales M.F., Girard L.-C., Raouna A., MacBeth A. (2023). The association of different presentations of maternal depression with children’s socio-emotional development: A systematic review. PLoS Glob. Public Health.

[144] Buchman A.L., Dubin M.D., Moukarzel A.A., Jenden D.J., Roch M., Rice K.M., Gornbein J., Ament M.E. (1995). Choline deficiency: A cause of hepatic steatosis during parenteral nutrition that can be reversed with intravenous choline supplementation. Hepatology.

[145] Zhao N., Yang S., Hu Y., Dong H., Zhao R. (2014). Maternal betaine protects rat offspring from maternal high-fat diet-induced visceral adiposity. J. Nutr. Biochem..

[146] Mehedint M.G., Niculescu M.D., Craciunescu C.N., Zeisel S.H. (2010). Choline deficiency alters global histone methylation and epigenetic marking at the Re1 site of the calbindin 1 gene. FASEB J..

[147] Uzunov A.V., Secara D.C., Constantin A.E., Mehedintu C., Cirstoiu M.M. (2022). Difference between Preterm Birth in Adolescent and Adult Patients. Maedica.

[148] Lees C.C., Marlow N., van Wassenaer-Leemhuis A. (2015). 2 year neurodevelopmental and intermediate perinatal outcomes in infants with very preterm fetal growth restriction (TRUFFLE): A randomised trial. Lancet.

[149] Zeisel S.H., Da Costa K.A. (2009). Choline: An essential nutrient for public health. Nutr. Rev..

[150] Bekdash R.A., Zhang C., Sarkar D.K. (2013). Gestational choline supplementation normalized fetal alcohol-induced alterations in histone modifications, DNA methylation, and proopiomelanocortin (POMC) gene expression in β-endorphin-producing POMC neurons of the hypothalamus. Alcohol. Clin. Exp. Res..

[151] King C., Plakke B. (2025). Maternal choline supplementation in neurodevelopmental disorders: Mechanistic insights from animal models and future directions. Nutr. Neurosci..

[152] Korsmo H.W., Dave B., Trasino S.E., Holscher H., Ivanov I., Stackhouse C., Saxena A., Jiang X. (2020). Prenatal choline supplementation during high-fat feeding improves long-term blood glucose control in male mouse offspring. Nutrients.

[153] World Health Organization (2024). Sexual, Reproductive, Maternal, Newborn, Child and Adolescent Health: Report on the 2023 Policy Survey.

[154] Jaiswal A., Dewani D., Reddy L.S., Patel A., Jindam A. (2023). Choline supplementation in pregnancy: Current evidence and implications. Cureus.

